# Gene expression differences between matched pairs of ovarian cancer patient tumors and patient-derived xenografts

**DOI:** 10.1038/s41598-019-42680-2

**Published:** 2019-04-19

**Authors:** Yuanhang Liu, Pritha Chanana, Jaime I. Davila, Xiaonan Hou, Valentina Zanfagnin, Cordelia D. McGehee, Ellen L. Goode, Eric C. Polley, Paul Haluska, S. John Weroha, Chen Wang

**Affiliations:** 1 Department of Health Sciences Research, Mayo Clinic, Rochester, MN 55905 USA; 2 Department of Oncology, Mayo Clinic, Rochester, MN 55905 USA; 3 Department of Obstetrics and Gynecology, Mayo Clinic, Rochester, MN 55905 USA

**Keywords:** Cancer genomics, Ovarian cancer, Computational biology and bioinformatics

## Abstract

As patient derived xenograft (PDX) models are increasingly used for preclinical drug development, strategies to account for the nonhuman component of PDX RNA expression data are critical to its interpretation. A bioinformatics pipeline to separate donor tumor and mouse stroma transcriptome profiles was devised and tested. To examine the molecular fidelity of PDX versus donor tumors, we compared mRNA differences between paired PDX-donor tumors from nine ovarian cancer patients. 1,935 differentially expressed genes were identified between PDX and donor tumors. Over 90% (n = 1767) of these genes were down-regulated in PDX models and enriched in stroma-specific functions. Several protein kinases were also differentially expressed in PDX tumors, e.g. PDGFRA, PDGFRB and CSF1R. Upon *in silico* removal of these PDX-donor tumor differentially expressed genes, a stronger transcriptional resemblance between PDX-donor tumor pairs was seen (average correlation coefficient increases from 0.91 to 0.95). We devised and validated an effective bioinformatics strategy to separate mouse stroma expression from human tumor expression for PDX RNAseq. In addition, we showed most of the PDX-donor differentially expressed genes were implicated in stromal components. The molecular similarities and differences between PDX and donor tumors have implications in future therapeutic trial designs and treatment response evaluations using PDX models.

## Introduction

Despite *in vitro* preclinical data to support the efficacy of many novel agents, translation to the clinic has been underwhelming^1,2^. Preclinical models are often limited in their ability to reflect the complexity and heterogeneity of patient tumors^2^. In addition, tumor cell lines can differ dramatically from the tumors from which they are derived^3^ and these discrepancies may increase over time, further limiting translation of findings to clinical practice^4^. Patient derived xenograft (PDX) models partially overcome limitations of cell-line based models and are widely used as preclinical models for drug development across many tumor types^5–9^.

In the context of ovarian cancer (OC) research, PDX models recapitulate key characteristics of the original donor tumor^10^. For instance, the donor tumor and corresponding PDX-tumor share similar histologic features including the extent of stromal infiltration, proliferation index measured by Ki-67, and expression of cytokeratins. Genomic aberrations of PDX tumors were also evaluated by array CGH, demonstrating faithful preservation of copy number changes^10,11^. Moreover, clinically relevant germline mutations in cancer-related genes, such as *BRCA1/2*, are persistent after multiple passages of PDX tumor *in vivo*^12^. However, other studies suggest that the genomic landscape of a PDX tumor can diverge from the donor tumor by clonal selection and/or clonal evolution^13,14^. This could give rise to differences in transcriptional activity, which has been reported in a breast cancer PDX-donor tumor pair^15^. Given the relevance of PDX models for preclinical drug development and potentially as a platform for personalized therapy, methods to assess human gene expression without interference by confounding nonhuman components are essential for proper interpretation of gene expression changes that occur as a result of xenotransplantation rather than the tested drug therapy.

Here we examined in detail the molecular differences of gene expression profiles between paired donor and PDX tumors using RNA sequencing (RNAseq). Since PDX tumor contains mouse stromal tissues, we devised and examined an effective bioinformatics pipeline to separate human-tumor and mouse-stroma transcriptome signals from PDX RNAseq. Differential expression analysis was conducted for nine pairs of donor-tumors and PDX tumors. In addition, differentially expressed genes between donor and PDX tumor pairs, likely representing the lack of human stromal elements in the PDX, were examined in a public OC dataset that used laser capture micro-dissection to separate ovarian carcinoma cells from the tumor stroma^16^. Possible reasons for the observed differences between donor PDX tumor pairs and potential implications for PDX study design and interpretation are discussed.

## Methods

### Total RNA extraction

Total RNA was isolated from tissues collected from patients and matched PDX from mice using the RNeasy Micro kit (Qiagen, #74004) according to the manufacturer instructions. Purity of total RNA and concentration was determined on a Thermo Scientific NanoDrop 2000c UV-Vis Spectrophotometer (Thermo Scientific, Wilmington, DE). All samples met RNA integrity number and validated Agilent (Agilent Technologies, Santa Clara, CA) criteria.

### mRNA library preparation and sequencing

RNA libraries were prepared according to the manufacturer’s instructions for the TruSeq RNA Sample Prep Kit (Illumina, San Diego, CA, USA). The concentration and size distribution of the libraries were determined on an Agilent Bioanalyzer DNA 1000 chip (Santa Clara, CA, USA). Libraries were loaded onto flow cells at concentrations of 8–10 pM to generate cluster densities of 700,000/mm2 following Illumina’s standard protocol using the Illumina cBot and cBot Paired End cluster kit version 3. The flow cells were sequenced as 51 × 2 Paired End reads on an Illumina HiSeq. 2000 using TruSeq SBS sequencing kit version 3 and SCS version 1.4.8 data collection software. Base calling was performed using Illumina’s RTA version 1.12.4.2. There were approximately 45 million reads per sample mapped to the human genome, and 21,686 genes were detected.

### Reverse-transcriptase quantitative PCR validation

To validate the RNAseq results, six top significantly-varied differentially-expressed genes (DEGs) between donor-PDX pairs were chosen for quantitative real-time PCR according to fold change and expression level by RNAseq: three down-regulated genes in PDX with a fold change of more than 5 (*FABP4*, *FAP* and *DCN*) and three up-regulated genes in PDX with a fold change of more than 1.5 (*PAX2*, *FOXB1* and *SBK2*). Between 300–500 ng of tissue RNA was reverse transcribed using ABI High capacity RNA to cDNA kit (Cat# 4387406; ThermoFisher Scientific, Waltham MA) as described in the manufacturer’s instructions and resulting cDNA was diluted 1:5 in molecular grade RNase/DNase free H2O. The quantitative real-time PCR was performed using the LightCycler 480-II with Lightcycler 480 Syber Green Master kit (Cat# 04707516001; Roche Diagnostic Ltd, Basel, Switzerland). The gene-specific primers were designed using the “Primer3 input” software (http://frodo.wi.mit.edu/primer3/), and their specificity was verified using the Primer-BLAST software (http://www.ncbi.nlm.nih.gov/tools/primer-blast/index.cgi?LINKLOC=BlastHome). GeneBank accession numbers of the six genes examined and their respective primer pair sequences are shown in Table S1. Quantitative PCRs were run and the melting curves of the amplified products were used to determine the specificity of the amplification. The threshold cycle number for the genes analyzed was normalized to RPLP0 as housekeeping gene. Fold changes between samples were determined using the ΔΔCt method. Data is presented as mean + Standard Error of Mean (SEM).

### Bioinformatics pipelines for processing donor- and PDX-tumors

**Figure 1:**
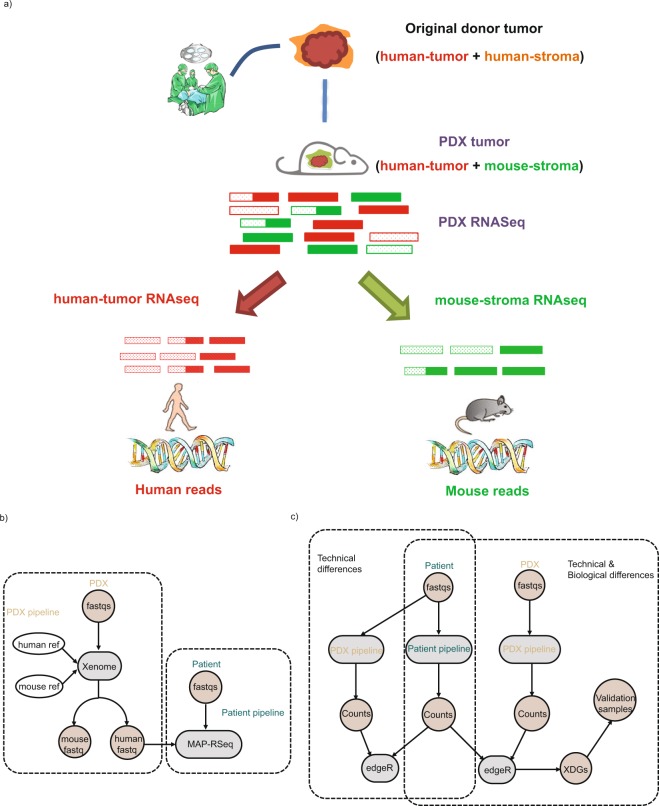
Illustration of bioinformatics strategy and workflows for analyzing patient- and PDX-RNAseq. (**a**) Bioinformatics strategy to separate mouse-stroma and human-tumor expression levels from PDX RNAseq data; (**b**) Dotted box indicates patient and PDX Pipelines for processing donor tumors and PDX tumors, respectively; (**c**) Dotted box indicates workflow for examining technical and biological differences.

For donor tumors’ RNAseq, the samples were processed with MAP-RSeq version 2.1.1 (Patient pipeline, Fig. 1b). MAP-RSeq uses a variety of publicly available bioinformatics tools tailored by in-house developed methods. Briefly, the aligning and mapping of reads is performed using TopHat2^17^ against the hg19 reference genome. The gene and exon counts are generated by FeatureCounts^18^ using the gene definitions files from Ensembl v78. All samples passed quality control according to RSeqQC^19^ and additional checks^20^. RNASeq data from all PDXs were first processed with Xenome (version 1.0.1) to classify the raw sequenced reads into human or mouse reads (PDX pipeline). Only the reads that were classified as ‘graft’, ‘ambiguous’ or ‘both’ were selected as the ‘human’ portion of PDX tumors. The ‘Human’ portion of PDX tumors was then processed through standard MAP-RSeq pipeline.

### RNAseq differential expression analysis

After removing genes with low expression (average counts per million across all samples smaller than 2), 16,968 genes were then tested for differential expression using edgeR paired analysis (version 3.20.1). In order to detect potential technical differences that might be due to different bioinformatics pipelines for donor tumors and paired PDXs, the same nine donor tumors were processed with both patient and PDX pipelines followed by differential expression analysis to identify differential genes caused by pipeline difference. Differential expression analysis for PDX versus matched donor-tumors was carried out using edgeR paired analysis to identify differentially expressed genes caused by engraftment process.

### Calculation of conservation score

Phast conservation scores were retrieved from UCSC genome database in bigwig format^21,22^. The conservation score for each gene was calculated based on average scores of all covered bases that belong to each gene.

### Examination of PDX-donor differential genes in independent human dataset

A publicly available, independent OC expression dataset that separately assessed the transcriptome profile of stroma- and carcinoma components from 31 patients^16^ was downloaded from GEO (GSE40595). Preprocess steps were carried out using affy (version 1.56.0). Differential testing between tumor-associated stroma and tumor epithelia cells was done using limma (version 3.34.0). A hypergeometric test was used to examine the significance of enrichment between PDX-donor differentially expressed genes and stroma-carcinoma differentially expressed genes (Yeung’s dataset).

### Gene set enrichment and association analysis

Gene set enrichment analysis was used to identify overrepresented biological functions for DEGs between PDX and donor tumor (XDGs) using GSEA software (version 3.0) and MsigDB database (version 6.1)^23^. Toppgene^24^ was used to examine biological functions enriched in genes overlapping between DEGs of PDX-donor pairs and DEGs of tumor-stroma/tumor-epithelium from the public dataset. Fisher’s exact test was adopted to examine the enrichment statistics of PDX-donor differentially expressed genes in several MsigDB defined gene families, including cell differentiation markers, cytokines and growth factors, homeodomain proteins, translocated cancer genes, oncogenes, protein kinases, transcriptional factors and tumor suppressors. Spearman correlation was used to examine expression similarity of XDGs with previously defined subtype signatures for ovarian cancer. Only overlapping genes between XDGs and subtype signature genes were considered for correlation analysis.

### Genotype calling for human donor tumor and PDX pairs

RVBoost^25^ was applied to identify genetic variants based on RNA sequencing data. Variants with an RVBoost q score smaller than 0.05 were removed from further analysis. BioR^26^ was used to annotate each identified variant with databases, such as dbSNP^27^ and COSMIC^28^. Sample genotype correlation was further evaluated using NGSCheckMate^29^.

### Generation of PDX models, ethical considerations, and informed consent

All studies with human samples were approved by Mayo Clinic Institutional Review Board (IRB). All animal studies were carried out in accordance with the relevant guideline and regulations of the Mayo Clinic Institutional Animal Care and Use Committee. All methods were performed in accordance with the relevant guidelines and regulations. Fresh tissues from nine patients with ovarian or fallopian tube cancer (eight serous and one clear cell) were collected at the time of primary debulking surgery at Mayo Clinic, Rochester. Written informed consent was obtained from all patients and documented in the electronic medical record. All tissues were coded with a patient heterotransplant (PH) number to protect patient identity in accordance with the Mayo Clinic IRB and in accordance with the Health Insurance Portability and Accountability Act through the Mayo Clinic Ovarian Tumor Repository. PDXs were developed by intraperitoneal injection of the donor tumor into female SCID beige mice (C.B.-17/IcrHsd-Prkdcscid Lystbg; ENVIGO). Briefly, 0.1 to 0.3 cc of grossly malignant tissue was minced and mixed 1:1 with McCoy’s media, supplemented with a one-time dose of Rituximab at the time of initial tumor implantation to reduce the occurrence of spontaneous lymphomas^30^, and injected intraperitoneally. Since the half-life of Rituximab in mice is only 5 days^31^ and CD20 expression is specific to B lymphocytes, the impact of this critical quality control step should not impact carcinoma transcriptomics when assessed months after initial injection and after multiple tumor passages without Rituximab re-treatment. No enzymatic or mechanical tumor dissociation was performed. Mice were monitored by routine palpation for engraftment and tumors were harvested when moribund. PDX models are reported here in accordance to the international minimal information standards^32^ (Table S2).

## Results

### RNAseq bioinformatics pipeline separating human-tumor and mouse-stroma transcriptomes

Since ovarian cancer PDX tissue is known to have murine stromal infiltrates^10^, RNAseq data from PDX tissue is expected to contain a mixture of human and mouse reads. A PDX RNAseq analysis pipeline was devised based on Xenome and a standard patient tumor pipeline (Fig. 1a,b). Although it is common practice to analyze PDX data by aligning sequencing reads to both human and murine reference genomes with subsequent filtering to identify murine reads, a careful validation of this practice to evaluate the accuracy of capturing human reads is needed. Accordingly, nine donor tumor samples, without prior passage in mice, were processed in the PDX pipeline to determine which reads were mis-identified as murine. The mapped ‘human’ reads were then submitted into the standard MAPR-Seq pipeline (Fig. 1c dashed box). In parallel, the same samples were processed through the standard patient pipeline to serve as a reference control. Subsequently, differential expression analysis was performed to examine the concordance of gene expression by pipeline as detailed in the Methods section. Any differentially expressed genes (DEGs) would reflect a technical artifact introduced by the PDX pipeline.

**Figure 2:**
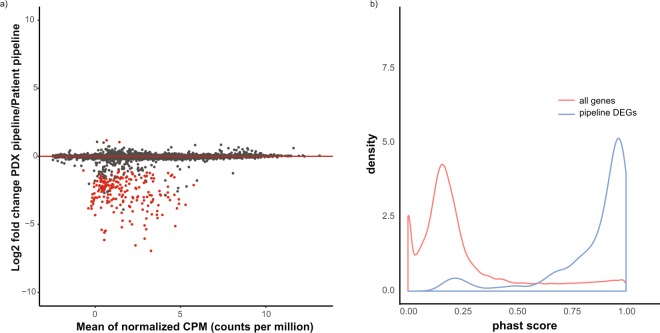
Identification of genes sensitive to patient- versus PDX-RNAseq bioinformatics pipelines. Nine patient donor tumors were processed through patient- and PDX-RNAseq piplines separately; differential expressed genes (DEGs) between the two pipelines are used to determine genes sensitive to pipeline differences. (**a**) MA (M: log ratio, A: Mean average) plot with DEGs highlighted in red; (**b**) Distribution of phast conservation score for all genes and DEGs caused by pipeline differences.

Expression profiles of the nine donor tumors measured by the two pipelines was highly concordant (average correlation coefficient 0.99) and only ~1% of DEGs (n = 215) were detected out of 16968 examined genes (FDR < 0.05; Absolute log2 fold-change > 1) (Fig. 2a and Table S3). Since a key step in the PDX pipeline is alignment to human and mouse genomes, we hypothesized that these false-positive DEGs are enriched in conserved genetic regions. These regions are expected to be highly similar between the two species, thereby affecting the read classification step in Xenome. To test this hypothesis, phast conservation scores, which are designed to reflect the degree of evolutionary conservation, were used. Indeed, DEGs due to pipeline differences are more conserved between human and mouse with a relatively high conservation score (mean phast score: 0.85, Fig. 2b). Since the 215 genes could not be reliably detected in the PDX pipeline as ‘human’, they were excluded from the following analyses.

### Transcriptomic differences between patient donor tumors and paired PDXs

**Figure 3:**
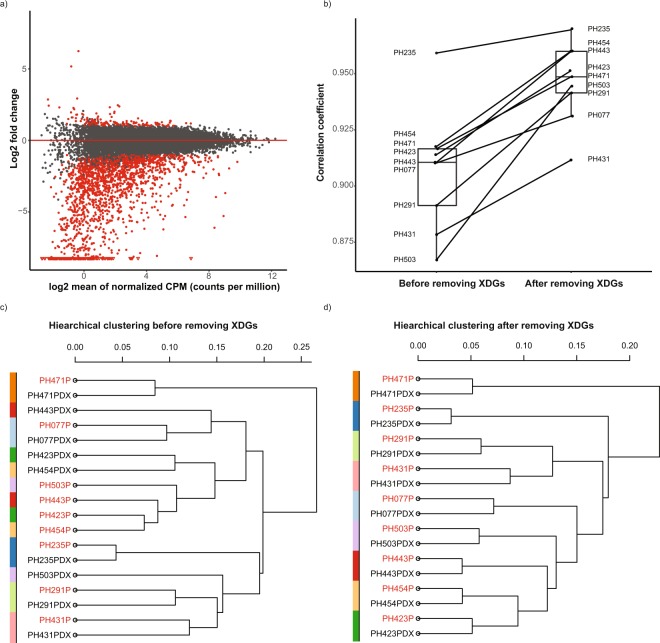
Expression differences of donor-PDX tumor pairs and impact on transcriptome pair similarity. RNASeq for nine pairs of donor/PDX tumors were processed with patient and PDX pipelines respectively. XDGs indicate differentially expressed genes between paired donor/PDX tumors after excluding previously identified genes that are sensitive to pipeline differences. (**a**) MA plot with XDGs in red; (**b**) Box plot of correlation coefficients of paired PDX-donor tumors before and after removing XDGs; (**c**) Hierarchical clustering of donor/PDX tumor pairs before removing XDGs; (**d**) Hierarchical clustering of donor/PDX tumor pairs after removing XDGs. Patient hetrotransplant (PH) numbers represent a single tumor line and the suffix indicates either the patient donor (P) or corresponding xenograft (PDX).

In order to examine transcriptional differences between paired patient donor tumor and PDX tumor, gene expression profiles for nine pairs of donor/PDX tumors were compared using RNAseq. All patient tumors were processed through the MAP-RSeq pipeline, and all PDX tumors were processed with the Xenome pipeline (Fig. 1b. dotted box). All nine PDX tumors exhibit a high percentage of reads uniquely mapped to the human reference genome (avg. 78.4%, Table S2) compared to the mouse genome (avg. 16.3%, Table S2). In total, 1935 DEGs were identified with absolute log2 fold change larger than 1 and FDR smaller than 0.05 after excluding the 215 previously identified artificially ‘differentially regulated’ genes (Fig. 3a and Table S3). These genes are referred to as PDX-Donor differential Genes (XDGs) since the primary biological difference between the paired tumors is the host within which the tumor resided at the time of RNA sequencing. The top three significantly up-regulated XDGs (up-XDGs) and three down-regulated XDGs (down-XDGs) were successfully validated using PCR (Fig. S2). Gene set enrichment analysis indicated that XDGs were enriched for up-regulation in cell growth and proliferation (cell cycle regulation FDR = 2e-3, DNA replication regulation FDR < 2e-16, etc.) and down-regulation in immunologic functions (immune response regulations, regulation of cell adhesions, etc., FDR < 2e-16) (Table S4). This is consistent with known replacement of human stroma with murine cells^10^ and loss of human immune infiltrates^30^ in the PDX.

To assess the potential impact of tumor passage on XDGs, a Spearman correlation coefficient of PDX gene expression with PDX passage was performed (Fig. S1). The distribution of correlation coefficients was symmetric (Fig. S1a) and the p values were largely uniformly distributed for all genes or XDGs separately (Fig. S1b,c). After multiple test correction, no genes are associated with passage at false discovery rate of 30%. This indicates that PDX gene expression is not associated or affected by PDX passage for our study.

To examine whether exclusion of XDGs would improve concordance between paired donor and PDX transcriptomes, the pair-wise correlation of donor-PDX pairs was compared before and after removing XDGs. Indeed, the average correlation coefficient of gene expression values in donor-PDX pairs was improved from 0.91 to 0.95 (Fig. 3b). In addition, unsupervised hierarchical clustering of donor-PDX pairs increased the proportion of aligned pairs from 5/9 (with XDGs) to 9/9 (without XDGs), leading to improved and unambiguous grouping of donor/PDX pairs (Fig. 3c,d). Not surprisingly, exclusion of the relatively small 215 previously identified artificially ‘differentially regulated’ genes had minimal influence over the correlation between donor tumor/PDX pairs since they represent only ~1% of transcripts (Table S5)_._

### Tumor microenvironment significantly accounts for donor/PDX transcriptomic differences

**Figure 4:**
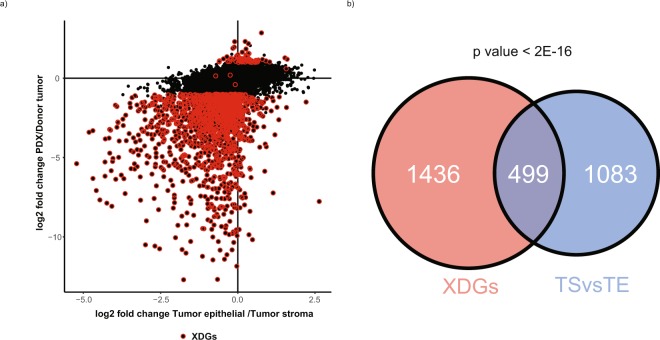
Comparing XDGs with human-stroma and -tumor expression profiles derived from laser micro-dissected tissues. An independent dataset (GEO#: GSE40595) that measures expression profile for laser micro-dissected tumor stroma and tumor epithelial tissues were downloaded and processed. (**a**) Pairwise scatter plot of the expression log2 fold changes: ovarian tumor versus stroma differences calculated from GEO#: GSE40595 (x-axis); PDXs versus donor tumors (y-axis); red dots indicate XDGs; (**b**) Venn diagram indicating overlapping genes across the two sets of differentially expressed genes: (i) XDGs and (ii) Tumor Stroma (TS) vs Tumor Epithelial (TE).

Since > 90% of XDGs were down-XDGs in PDX tumor and enriched for stroma-related pathways, the hypothesis that XDGs are predominately due to the loss of human stroma in PDX tissues was further examined using a public OC expression dataset derived from tumors in which the RNAseq was separately performed on epithelial carcinoma or the surrounding stromal components. By re-analyzing this public dataset, 1582 DEGs were identified between tumor-epithelial (TE) and tumor-associated stroma (TS) components with a fold-difference comparable to our dataset of PDX vs donor tumor when the PDX analysis was restricted to human carcinoma transcripts and donor tumor analysis included carcinoma plus stroma transcripts (cor = 0.39, FDR < 2e-16) (Fig. 4a). When comparing stroma-tumor differentially expressed genes from the public data and XDGs from the PDX-donor analysis above, 499 overlapping genes were found with significant enrichment (FDR < 2e-16; odds ratio = 3.8) (Fig. 4b). Functional annotation of these genes indicated that they were highly enriched for immune related genes and extracellular matrix (ECM) related functions (Table S6), (e.g. immune response (FDR = 6e-12) and cell adhesion (FDR = 3e-27)). Intriguingly, the non-overlapping XDGs are also strongly enriched in biological functions (Table S7) such as immune response (FDR = 9e-29) and cell adhesion (FDR = 2e-18). Those genes may reflect gene expression changes required for a human tumor to thrive in a murine host.

### Gene Set Enrichment Analysis for Identified XDGs

To explore possible implications of XDGs in preclinical drug development, a series of gene set enrichment or association analyses were conducted with different cancer-related gene categories (Table S8), targetable protein kinases (Table S9), and previously reported gene signatures defining various ovarian cancer molecular subtypes (Table S10). In particular, down-XDGs were over-represented in genes known to be cell-differentiation markers (p value = 1.38e-62, Table S8). XDGs contain 37 known protein kinase coding-genes, including many clinically evaluated genes, e.g. PDGFRA, BTK, and JAK2. When the list of kinase XDGs was cross-referenced with a diverse set of 72 kinase inhibitor compounds^33^, all genes matched to at least 2 compounds with a binding result less than 300 nM. Twenty-nine of those protein kinases were previously shown to be targetable by at least five compounds (Table S9)^33^. In addition, XDGs significantly correlated with the so-called “mesenchymal” molecular subtype of ovarian tumors (correlation coefficient = −0.42, p = 6.5e-3 for Wang’s subtype signatures; correlation coefficient = −0.29, p = 2.5e-12 for Tothill’s subtype signatures; Table S10, Figs S3 and S4), which was linked to poor survival and unfavorable surgical outcomes^34,35^.

### Genotype concordance between patient donor tumors and paired PDXs

Although genotype analysis was not a focus of this study, demonstrating concordance between donor and PDX tumor pairs is relevant to the assessment of PDX tumors as surrogates for primary tumors. In order to demonstrate the genomic fidelity of donor tumor/PDX pairs, we applied RVBoost to call genetic variants based on RNA sequencing data. After proper variant filtering and annotation, we found that donor tumor/PDX pairs indeed shared a significant portion of variants annotated in either dbSNP or COSMIC databases (Fig. S5a,b). We then evaluated pairwise sample genotype correlation using NGSCheckMate. As shown in Fig. S5c, the donor tumor/PDX pair relationship was correctly identified for all 9 pairs. This confirms that the genetic fidelity between donor tumor/PDX pairs is largely preserved.

## Discussion

Although ovarian cancer PDX models recapitulate key histologic characteristics and chemotherapeutic responses of the primary patient tumor, a detailed evaluation of the molecular differences between patient donor tumors and corresponding PDX models is needed to better understand the strengths and limitations of this technology. By measuring the transcriptional signature of paired donor/PDX tumor samples, we identified a relatively small percentage of genes affected by technical limitations of the bioinformatics pipelines (n = 215), which can be largely attributed to highly conserved human and murine genes. Importantly, 1935 genes exhibited significant differences between donor tumors and paired PDX tumors, which we termed as XDGs. After excluding XDGs, PDX tumors became more similar to the original donor tumors. Moreover, the categorization of XDGs as predominantly stromal was supported by comparisons with an independent external dataset.

Our study provides the first comparisons of ovarian donor tumors and corresponding PDX tumors using RNAseq. Several studies have also provided insights into the molecular differences between donor tumors and PDX tumors for other types of cancer^36–38^. However, most of the studies used hybridization-based microarray technologies for transcriptional profiling, which may have limited resolution to separate the molecular contributions from the murine host and human tumors. In comparison, by using RNAseq, we could separate the mouse component from PDX tumors by aligning to both human and mouse reference genomes *in-silico*. The 1935 XDGs that we identified were enriched in various biological processes, such as immune response and cell adhesion regulations. In addition, XDGs contain important gene families that are potentially targetable, e.g. 45 oncogenes and 48 kinases (Tables S3 and S8) as defined in the Molecular Signatures Database (MSigDB)^23^. Accordingly, PDX models may be well suited for preclinical studies with novel therapies, but interpretation of studies targeting XDGs should consider whether the target gene expression is truly a driver of malignancy or consequence of xenotransplantation.

This study implicates two contributing factors leading to the observation that XDGs appear “down-” or “up-regulated” in PDX tissue compared to the original patient tumors: 1) an adaptive response to environmental changes during engraftment (e.g. over-representation of oncogenes in XDGs, p value = 5.5e-3, Table S8) and 2) a loss of human immune and stromal cells in PDX tumor (over-representation of cell-differentiation markers in down-XDGs, p value = 1.38e-62, Table S8). However, the biological significance of up- or down-XDGs should be determined by individual investigators on a case-by-case basis and consider its implications on the design and interpretation of PDX *in vivo* data. For instance, cyclin D2 (CCND2) expression in our PDX cohort has a −4.771 log2 fold change expression compared to the primary donor tumor (down-XDG), indicating marked downregulation in the PDX. Accordingly, it is possible that the efficacy of inhibitors such as ribociclib (targeting CDK4/6, the activated binding partner of CCND2) may be underestimated in ovarian cancer PDX models with down-regulated CCND2 expression. However, since PDX models are often chosen based on pre-determined expression of a putative biomarker or expression of known target genes, the impact of down-XDGs may be minimized because low-expressing tumors would be excluded from investigation. In addition, it may be relevant for investigators to explore whether murine ligands can activate human receptor tyrosine kinases and whether receptor activation status impacts its own expression through feedback loop mechanisms.

Although the focus of this study was on transcriptomic profiling, fidelity of genetic and genomic alterations in PDX tumors are relevant to the broader argument that PDX tumors retain key molecular characteristics of the primary donor tumor. Even though copy number alterations (CNA) are largely conserved across several PDX generations by array comparative genomic hybridization^11,39^, an indirect computational algorithm to infer copy number alterations based on gene expression found noticeable CNA that may affect treatment responses^14^. For that specific study, due to the lack of DNA copy number data, the authors adopted a computational algorithm to infer copy number alterations from a gene expression dataset and successfully demonstrated the effectiveness of their approach. This approach, if validated more extensively with DNA copy number data and applied properly, potentially will benefit future PDX studies. Given that the current study did not focus on DNA changes, nevertheless, an exploratory analysis of genotype extrapolated from mRNA sequence showed high correlation between donor tumor and PDX, suggesting that individual gene alterations may be conserved. This has relevance for some therapeutic agents, such as poly (ADP-ribose) polymerase (PARP) inhibitors for ovarian cancers with *BRCA1/2* mutations^40–42^. However, some degree of genetic evolution is expected in higher-passage PDX tumors and conservation of specific aberrations should be confirmed prior to PDX model selection, if relevant to the specific investigational agent. Despite the differences between donor and PDX, it is not clear if XDGs would impact the predictive potential of cytotoxic chemotherapies; correlative studies from an ongoing PDX-directed therapy trial (MC1463, clinicaltrials.gov # NCT02312245) may be revealing.

The inclusion of a stage I clear cell histologic subtype (PH471) is in response to the growing call for novel therapies to treat patients who might otherwise be excluded from clinical trials or lack clinical trial options specifically for clear cell histology due to the rare nature of this disease^43^. Since most patients with clear cell ovarian cancer are diagnosed at an early stage, models like PH471 address an unmet need.

Limitations in the current study are recognized. For instance, donor/PDX tumor molecular differences may be partially attributed to clonal selection and evolution in mice^13^. Also, inflammatory cell components are lacking in current PDX models due to the immunodeficiency of the host mice. Importantly, the significance of identified XDGs with regard to drug response requires laboratory and clinical correlation to fully understand the nature and extent of influence on drug response.

In summary, standard bioinformatics pipelines to analyze PDX tumor RNA expression data are influenced by highly conserved human and mouse genes. Although some of the expression differences between donor and PDX tumor can be attributed to the inherent lack of human stroma in PDX tumor tissue, other differences may be secondary to expression of growth and pro-survival factors that permit xenotransplantation. As PDX models have become an important tool for preclinical drug development, such factors should be considered during study design and interpretation.

## Supplementary information

supplementary figures

supplementary tables

## Data Availability

Datasets and used in this study are available from GitHub at: https://github.com/Liuy12/PDX_paper.
