# Supplementary material for: Gene expression differences between matched pairs of ovarian cancer patient tumors and patient-derived xenografts
Source: Sci Rep. 2019 Apr 19;9:6314. doi: 10.1038/s41598-019-42680-2 (PMC6474864; doi:10.1038/s41598-019-42680-2)
Supplement: Supplementary file 1 — supplementary figures [file 41598_2019_42680_MOESM1_ESM.pdf]

Gene expression differences between matched pairs of ovarian cancer patient tumors and patient-derived xenografts

Yuanhang Liu, Chanana Pritha, Jaime I. Davila, Xiaonan Hou, Valentina Zanfagnin, Cordelia D. McGehee, Ellen L. Goode, Eric C. Polley, Paul Haluska, S. John Weroha, Chen Wang

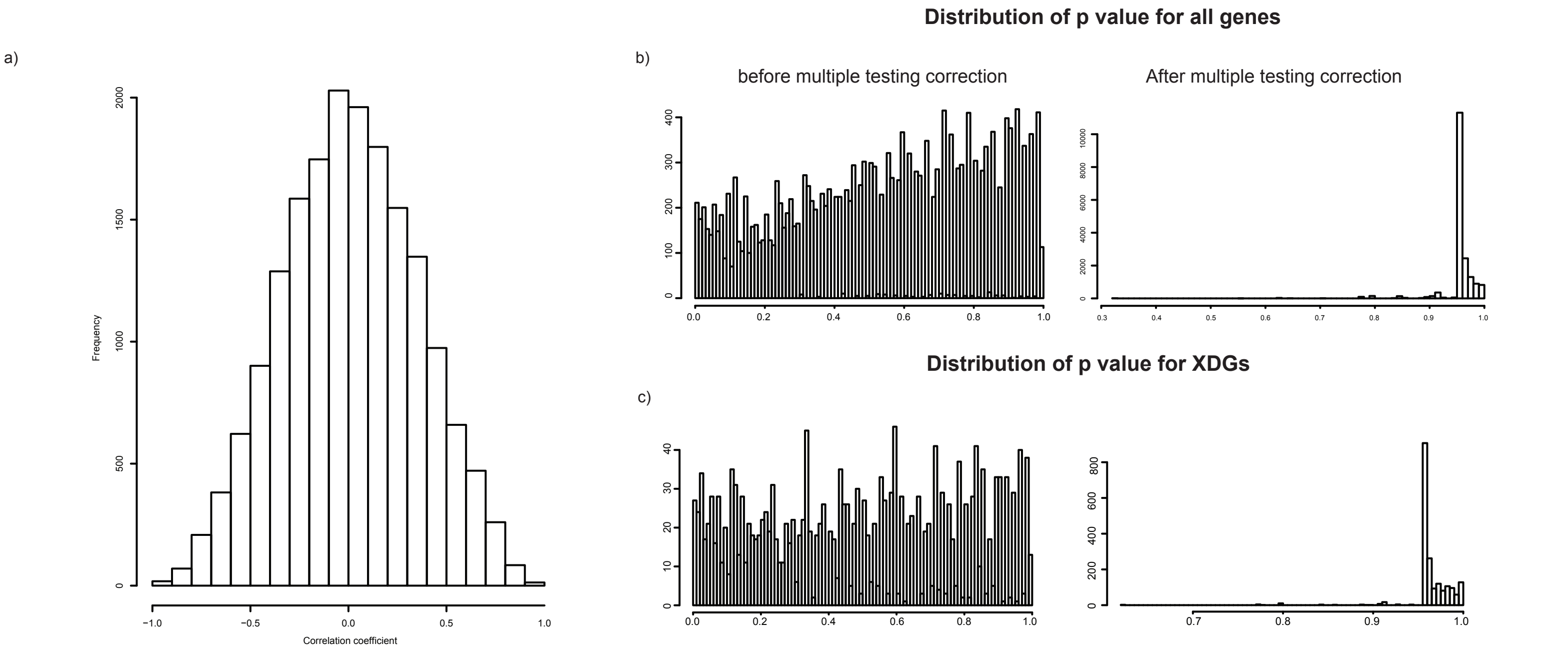

Figure S1. (a) Distribution of correlation coefficients between PDX passage and gene expression. (b) Distribution of p-values associated with correlation coefficients for all genes. (c) Distribution of p-values associated with correlation coefficients for XDGs.

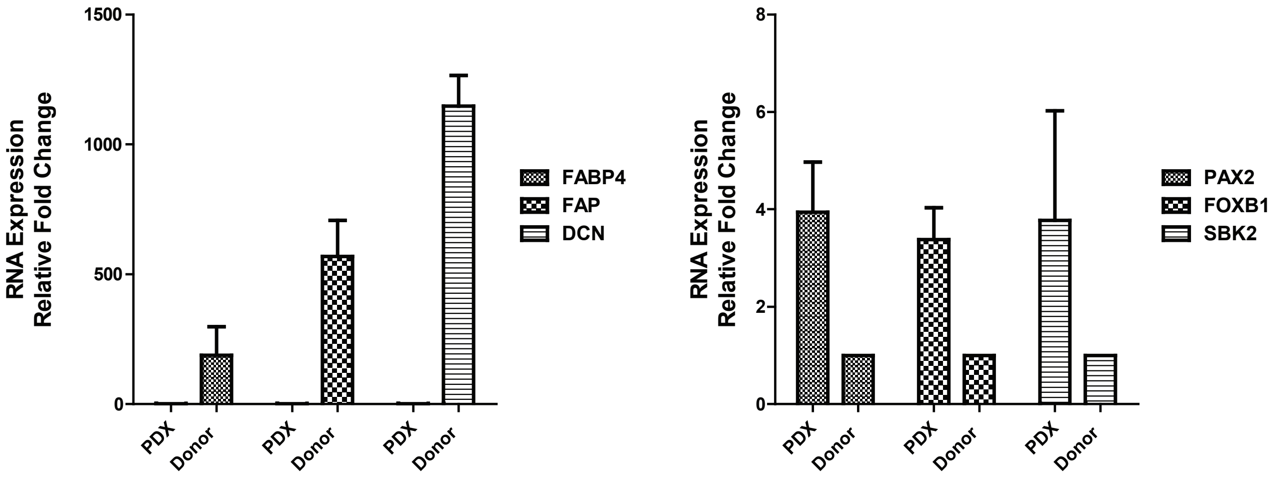

Figure S2. RT-qPCR validation of (a) three up-XDGs and (b) three down-XDGs. Data presented as relative expression fold change between PDX and Donor tumor. Error bars represent standard error of the mean (n = 3). Our qRT-PCR results indicate that expression of these 6 genes is consistent with our RNA-seq results.

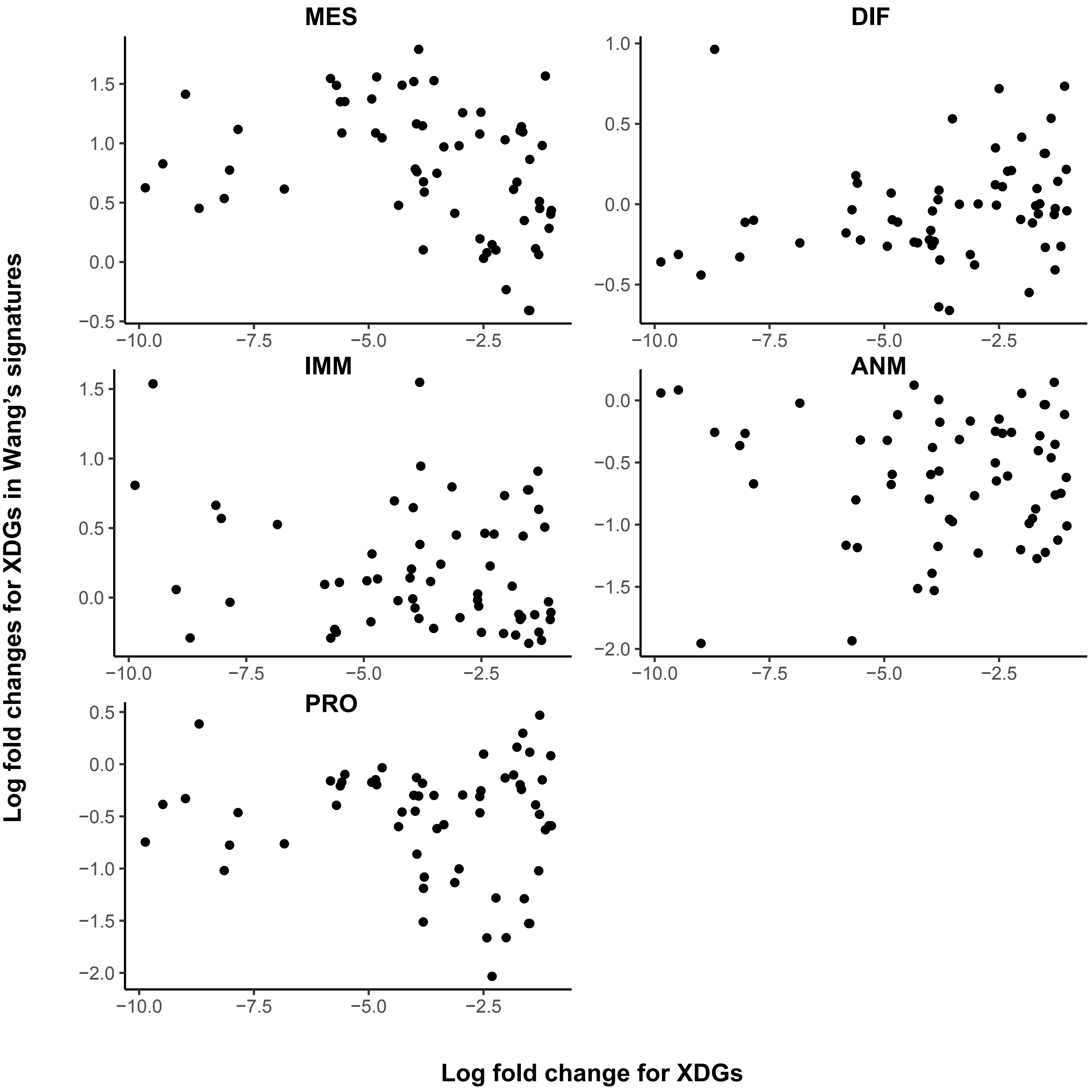

Figure S3. Comparison of XDGs with ovarian cancer subtype signatures defined by Wang. x axis indicates log2 fold change of XDGs for PDX/donor comparison. y axis indicates log2 fold change of XDGs for ovarian cancer subtype signatures defined by Wang.

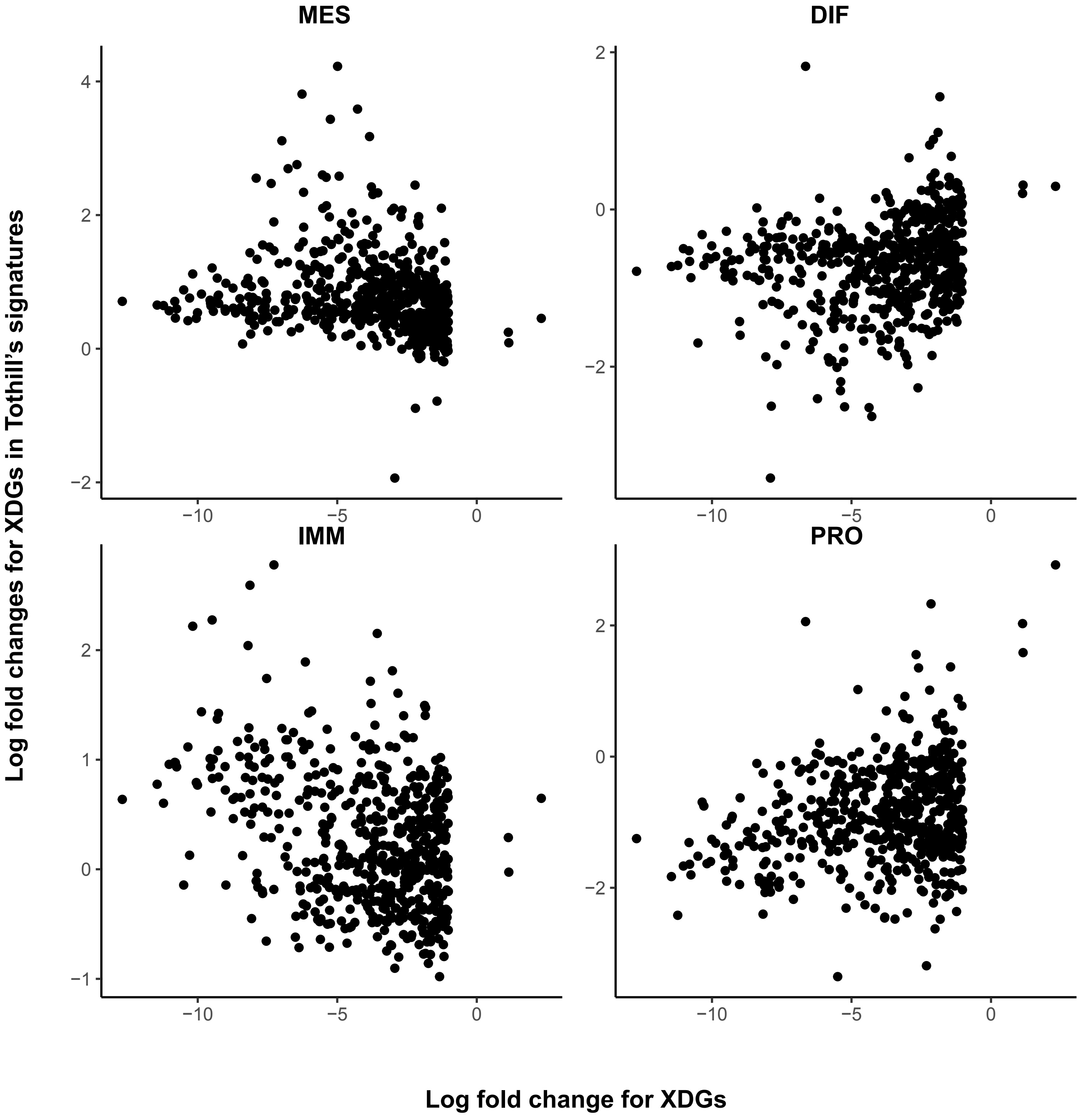

Figure S4. Comparison of XDGs with ovarian cancer subtype signatures defined by Tothill. x axis indicates log2 fold change of XDGs for PDX/donor comparison. y axis indicates log2 fold change of XDGs for ovarian cancer subtype signatures defined by Tothill.

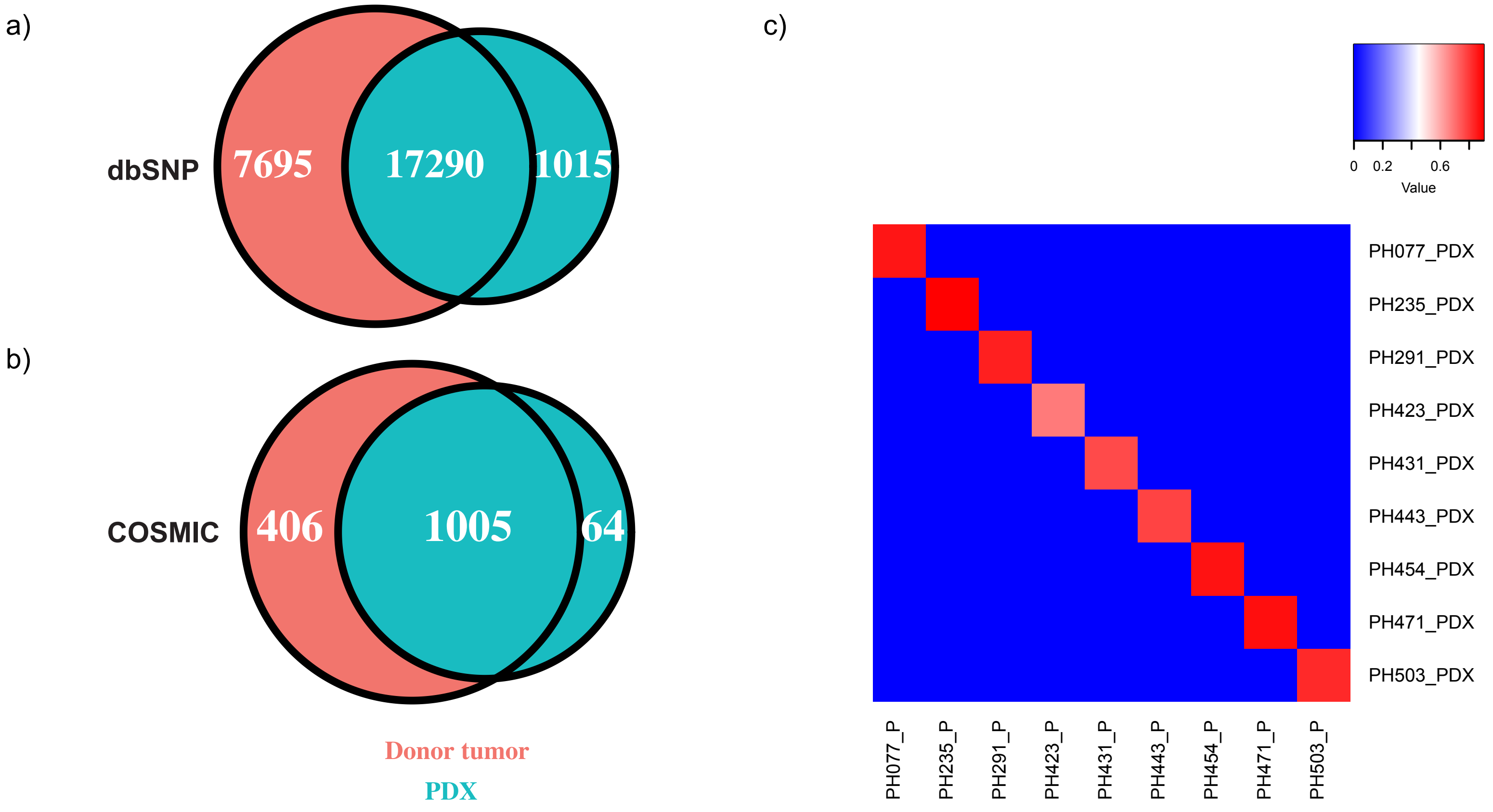

Figure S5: Venn diagram comparing overlap of a) dbSNP annotated; b) COSMIC annotated variants between donor tumor and PDX for PH503; c) Genotype concordance between donor tumor/PDX pairs examined using NGSCheckMate.
